# Step and Distance Measurement From a Low-Cost Consumer-Based Hip and Wrist Activity Monitor: Protocol for a Validity and Reliability Assessment

**DOI:** 10.2196/21262

**Published:** 2021-01-13

**Authors:** Thomas Carlin, Nicolas Vuillerme

**Affiliations:** 1 AGEIS University Grenoble Alpes Grenoble France; 2 LabCom Telecom4Health University Grenoble Alpes & Orange Labs Grenoble France; 3 Institut Universitaire de France Paris France

**Keywords:** activity monitor, pedometer, measurement, validity, reliability, walking, step count, distance

## Abstract

**Background:**

Self-tracking via wearable and mobile technologies is becoming an essential part of personal health management. At this point, however, little information is available to substantiate the validity and reliability of low-cost consumer-based hip and wrist activity monitors, with regard more specifically to the measurements of step counts and distance traveled while walking.

**Objective:**

The aim of our study is to assess the validity and reliability of step and distance measurement from a low-cost consumer-based hip and wrist activity monitor specific in various walking conditions that are commonly encountered in daily life. Specifically, this study is designed to evaluate whether and to what extent validity and reliability could depend on the sensor placement on the human body and the walking task being performed.

**Methods:**

Thirty healthy participants will be instructed to wear four PBN 2433 (Nakosite) activity monitors simultaneously, with one placed on each hip and each wrist. Participants will attend two experimental sessions separated by 1 week. During each experimental session, two separate studies will be performed. In study 1, participants will be instructed to complete a 2-minute walk test along a 30-meter indoor corridor under 3 walking speeds: very slow, slow, and usual speed. In study 2, participants will be required to complete the following 3 conditions performed at usual walking speed: walking on flat ground, upstairs, and downstairs. Activity monitor measured step count and distance values will be computed along with the actual step count (determined from video recordings) and distance (measured using a measuring tape) to determine validity and reliability for each activity monitor placement and each walking condition.

**Results:**

Participant recruitment and data collection began in January 2020. As of June 2020, we enrolled 8 participants. Dissemination of study results in peer-reviewed journals is expected in spring 2021.

**Conclusions:**

To the best of our knowledge, this is the first study that examines the validity and reliability of step and distance measurement during walking using the PBN 2433 (Nakosite) activity monitor. Results of this study will provide beneficial information on the effects of activity monitor placement, walking speed, and walking tasks on the validity and reliability of step and distance measurement. We believe such information is of utmost importance to general consumers, clinicians, and researchers.

**International Registered Report Identifier (IRRID):**

DERR1-10.2196/21262

## Introduction

Self-tracking via wearable and mobile technologies is becoming an essential part of personal health management [1,2]. In recent years, wearable devices such as those made by Fitbit (eg, One, Flex, Ultra) and ActiGraph (GT9X, GT3X) have been widely introduced into the consumer market as physical activity monitors. The relatively low cost, interface capabilities, ease of use, and wide commercial availability of these devices may ultimately change the way researchers and clinicians alike monitor their patients’ physical activity [3,4] by providing remote access to patient-generated data [5]. In particular, Fitbit [6-14] and ActiGraph [15-25] trackers have received considerable attention.

Depending on the type of activity monitor, companies recommend wearing them at the waist, wrist, pocket, hip, or bra. These wearables devices contain different tools for measurement such as piezoelectric pedometers, single triaxial accelerometers, or inertial measurement unit that combine accelerometers, gyroscopes, and sometimes magnetometers. Using proprietary algorithms, data from measures collected along with information input by the user can estimate steps, distance, physical activity, kilocalories, and sleep [4,5,25,26]. Among these outputs, step and distance measurements while walking remain the most popular and translatable outputs in use today [4,25,27-29].

At this point, it is important to recall that valid and reliable step count and distance estimates are vital output metrics and constitute crucial selection criteria for use in clinical practice [28]. However, little information is available to substantiate the validity and reliability of step and distance measurement from consumer-based hip and wrist activity monitors [26,30,31]. More specifically, despite the increasing number of published works on the evaluation of the most well-known activity monitors [17,25,27,32,33], only a few addressed low-cost activity monitors. For instance, the GT3X (ActiGraph LLC), one of the most well-known and studied accelerometer in research and clinical setting [6-14], costs around €225 (US $250) for one device [34] plus the price of the software and accessories. Fitbit activity trackers also have been the subject of many studies [15-25]; for example, the Charge (Fitbit Inc) costs around €150 (US $170) [35]. This observation is all the more relevant as the selling price is a main barrier to purchase [36]. Indeed, numerous activity monitors available on the market are rather expensive and beyond the financial means of a significant segment of society.

Within this context, the aim of our study is to assess the validity and reliability of step and distance measurement from a low-cost consumer-based hip and wrist activity monitor specific to various walking conditions commonly encountered in daily life. This study is designed to evaluate whether and to what extent validity and reliability of step and distance measurement from a consumer-based hip and wrist activity monitor could depend on the sensor placement on the human body and the walking task being performed. Human locomotion in daily life involves walking at non–self-selected speeds, walking upstairs, walking downstairs, turning... in other words, not just straight line walking on flat ground at a comfortable speed. While reliability and validity of activity monitors for walking on flat ground is quite well documented in the growing literature on the subject [20,23,26,37-54], ascending and descending stairs has received less attention [20,23,44-48,50,51,53,55].

## Methods

### Participants

Young healthy adults aged 18 to 40 years will voluntarily participate to this work. Participants will be recruited in the Grenoble area (France) through open recruitment and direct invitation. From previous similar studies, we have identified that a minimum of 30 participants will be necessary to demonstrate significant differences between the experimental settings [38,47,56,57]. Participants cannot have any history of injury, surgery, or pathology to lower extremities that affects their gait.

The following participant demographic and anthropometric data will be collected: gender, age, body height, body weight, foot length, dominant leg, dominant arm, leg length, arm length, and physical activity level.

Each participant will sign a written informed consent prior to their participation in compliance with the Declaration of Helsinki. This study was approved by the local ethics committee (CER Grenoble Alpes, Avis 2019-04-09-4).

### Materials

#### Activity Monitors

The PBN 2433 (Nakosite USA Ltd) activity monitor has been selected according to previous recommendations that an activity monitor should cost less than €150 (US $185), require no monthly costs for a subscription, provide real-time feedback to the user, and have no chest strap for heart rate measurements [58,59].

The PBN 2433 is a small, inexpensive activity monitor (dimensions: 12 mm × 8 mm × 4 mm; weight: 31.2 g; €15 [US $20]) worn on a wrist band or clipped on the hip. The PBN 2433 tracks step count (total steps per day), calories burned (kcal/day), distance traveled (km/day), and exercise time (h/day). A multiple LCD screen displays direct feedback on outputs. No Bluetooth connection, supplementary app, or mobile or smartphone are needed. It sets up with weight (kg) and stride length (cm). Weight will be obtained by asking participant about their approximative weight. For stride length measurement, participant will be asked to walk 10 steps and the distance traveled will be divided by 10 to obtain an average stride length as is recommended by the manufacturer.

During all experiments, participants will be asked to wear four PBN 2433 activity monitors simultaneously in these locations on their body:

- Fitted on the right hip, positioned over the right anterior iliac spine via the manufacturer-provided silicone clip
- Fitted on the left hip, positioned over the left anterior iliac spine via the manufacturer-provided silicone clip
- Fitted to the right wrist using the manufacturer-provided wristband and positioned on the dorsal aspect of the wrist just proximal to the radial and ulnar processes
- Fitted to the left wrist using the manufacturer-provided wristband and positioned on the dorsal aspect of the wrist just proximal to the radial and ulnar processes

#### Video Processing

Participants will also be videorecorded with an embedded camera (HERO4, GoPro Inc) fixed on the chest thanks to the dedicated harness. The camera lens will be oriented toward the ground in order to capture all steps taken by the participant. In other words, as previously done by others [19,22,23,30,38,43,45,56,58,60-64], we will use the video-based step count as the gold standard for actual step count. Video-based step counting will be independently conducted by two observers, with a third observer repeating the count in case of discrepancy between obtained step counts [27,28,65,66].

### Experimental Procedure

Participants will attend two similar experimental sessions in an indoor environment separated by 1 week. During each experimental session, two different experiments will be conducted.

In experiment 1, as was previously proposed in published studies designed to evaluate the validity of activity monitors for measuring step counts and distance traveled while walking [18,20,24,54], participants will be required to complete a standard 2-minute walk test during which they will be videotaped. This walking task will be performed in an enclosed, wide, long, flat, 30-m corridor with the instruction to walk back and forth on the course all throughout the 2-minute period. This walking task will be performed under 3 walking speed [67]: (1) very slow walking speed, with the instruction “Walk very slowly”; (2) slow walking speed, with the instruction “Walk a little faster, but slower than normal”; and (3) usual walking speed, with the instruction “Walk at your normal, preferred speed.”

In experiment 2, participants will be videotaped during the walking task performed at usual walking speed under 3 conditions [68,69]: walking on flat ground, walking upstairs, and walking downstairs.

During the walking on flat ground condition, participants will complete a standard 2-minute walk test as proposed in experiment 1. During the walking upstairs condition, participants will be asked to ascend flights of stairs located inside a building stairwell for 2 minutes. During the walking downstairs condition, participants will be asked to descend flights of stairs located inside a building stairwell for 2 minutes. At this point, it is important to mention that handrails do represent important safety features to assist users to maintain their balance and prevent falls on stairs. However, for methodological reasons, participants will not be permitted to use handrails on stairs in the walking upstairs and walking downstairs experimental conditions.

During each experimental session and for each experiment, the order of the 3 experimental conditions will be randomized over participants to reduce potential carryover effects. Only one 2-minute walking trial per condition will be performed. Before to the formal walking trial, participants will complete a practice trial to familiarize themselves with the walking task. A 2-minute rest period will be given between each walking trial in order to record the step and distance counts from each activity monitor. During this period, participants will be instructed to stand still. The number of steps and walking distance measured by each activity monitor will be noted from the tracker display before and immediately after the completion of each trial.

### Statistical Analysis

Descriptive statistics and their corresponding 95% confidence intervals will be determined for all dependent variables. Step count and walking distance errors from each activity monitor will further be calculated as follows:

- *Step count error = [(steps measured – actual steps) / actual steps] x 100%*
- *Distance error= [(distance measured – actual distance) / actual distance] x 100%*

where:

- Steps counted and distance measured are the value of steps and distance provided by the activity monitor
- Actual steps is the number of steps manually counted from video
- Actual distance is the distance measured using a measuring tape

An error score of zero indicates no difference; a positive error score represents an overestimation of the step and distance counts; a negative error score represents an underestimation.

Validity will be determined by comparing the activity monitor outputs (step and walking distance) with the criterion measures (ie, step count determined from video recordings and distance measured using a measuring tape), using mean differences, mean absolute percentage errors (MAPE), and intraclass correlation coefficients (ICC). According to Feito et al [70,71], a MAPE exceeding 5% can be considered as a practically relevant difference. Repeated-measures analysis of variance (ANOVA) will be used to determine whether a significant difference exists between validity of activity monitor placements and walking conditions. In cases when ANOVA shows a significant difference, post hoc analysis will be performed via Bonferroni tests or, when variances is not assumed to be equal, via Games-Howell tests.

The correlation and level of agreement of the steps and distances estimated by the activity monitor to the criterion measures will be further assessed by calculating a Spearman correlation coefficient or Pearson correlation coefficient (*r*) and ICC, respectively. The parametrical or nonparametrical correlation formula is selected based on the linearity of the relationship between the two measured variables. A correlation coefficient value from .00 to .20 is considered poor, .20 to .40 fair, .40 to .60 moderate, .60 to .80 substantial, and .80 to 1.00 almost perfect [72]. An ICC value from .00 to .40 is considered poor, .40 to .59 fair, .60 to .74 good, and .75 to 1.00 excellent [73]. Bland-Altman plots will further be constructed to visually assess agreement of activity monitor estimates with the criterion measures [74].

Between-day reliability will be determined by calculating ICC between results obtained during session 1 and session 2. We will also compute the mean differences, standard error of measurements, and the MAPE between both experimental sessions. Significant differences between session 1 and session 2 will be determined by paired-sample *t* test or its nonparametric equivalent, the Wilcoxon signed-rank test. A Bland-Altman plot will be constructed to analyze the agreement between the two assessments.

Statistical significance will be set a priori at *P*<.05. All statistical calculations will be completed using the R software environment version 3.1.0 (R Foundation for Statistical Computing).

## Results

Participant recruitment and data collection began in January 2020. As of June 2020, we enrolled 8 participants. Dissemination of study results in peer-reviewed journals is expected in spring 2021.

## Discussion

Physical activity is now widely recognized as crucial for health status maintenance [75-78], whereas physical inactivity has been identified as one of the most important factors in the rise of noncommunicable diseases [78,79]. For these reasons, regular daily physical activity is fully recommended [41,80,81]. It is known that real-time monitoring of physical activity can lead to a perceptible improvement of the physical activity level [2,82-84]. Various activity monitors are available on the market for this purpose. Most of them are designed to measure steps and distance traveled while walking [4,5,26,27]. Although their beneficial effect on health is now well documented [2,82-84], little information is available to substantiate the validity and reliability of step and distance measurement, especially from low-cost consumer-based hip and wrist activity monitors.

To the best of our knowledge, this is the first study that examines the validity and reliability of step and distance measurement during walking conditions using the PBN 2433 activity monitor. To achieve this goal, young healthy participants will be asked to wear four PBN 2433 activity monitors simultaneously, placed on each hip and each wrist. Participants will be also instructed to perform various walking conditions that are commonly encountered in daily life during two experimental sessions.

On the whole, considering the existing literature on the topic, we hypothesize that the validity and reliability of step count and distance output from the PBN 2433 activity monitors will depend on the following factors:

- Sensor placement locations on the user’s body [21,38,46,51,85,86]
- Walking speed [38,55,87-91]
- Walking tasks being performed [23,46,50,55]

Taken together, these will provide beneficial information on the effects of activity monitor placement, walking speed, and walking tasks on validity and reliability of step and distance measurement. We believe that such information is of utmost importance to general consumers, clinicians, and researchers.

## Abbreviations

ANOVA: analysis of variance
ICC: intraclass correlation coefficient
MAPE: mean absolute percentage error
